# Supplementary material for: Integrated analysis of the aqueous humor microbiome and lens capsule transcriptome in high myopia cataract: a pilot study
Source: Front Med (Lausanne). 2026 Jun 16;13:1845205. doi: 10.3389/fmed.2026.1845205 (PMC13314463; doi:10.3389/fmed.2026.1845205)
Supplement: Supplementary file 3 [file Data_Sheet_1.zip › 6.PICRUSt2/Meta_Statistic/C372089/Wilcoxon/KEGG_L3.Wilcoxon.diff.top10.boxplot.pdf]

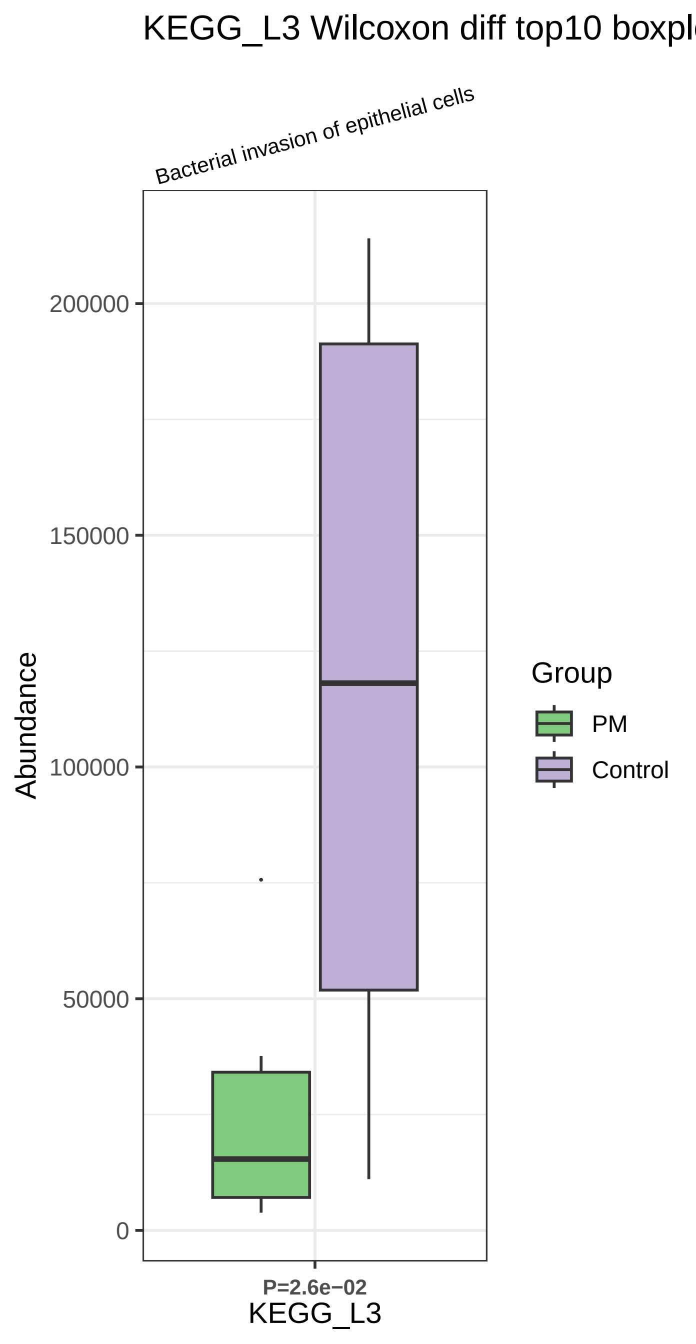
KEGG_L3 Wilcoxon diff top10 boxpl
Bacterial invasion of epithelial cells
Abundance
200000
150000
100000
50000
0
P=2.6e−02
KEGG_L3
Group
PM
Control
